# The P Body Protein Dcp1a Is Hyper-phosphorylated during Mitosis

**DOI:** 10.1371/journal.pone.0049783

**Published:** 2013-01-02

**Authors:** Adva Aizer, Pinhas Kafri, Alon Kalo, Yaron Shav-Tal

**Affiliations:** The Mina and Everard Goodman Faculty of Life Sciences and Institute of Nanotechnology, Bar-Ilan University, Ramat Gan, Israel; Institut de Génétique et Développement de Rennes, France

## Abstract

Processing bodies (PBs) are non-membranous cytoplasmic structures found in all eukaryotes. Many of their components such as the Dcp1 and Dcp2 proteins are highly conserved. Using live-cell imaging we found that PB structures disassembled as cells prepared for cell division, and then began to reassemble during the late stages of cytokinesis. During the cell cycle and as cells passed through S phase, PB numbers increased. However, there was no memory of PB numbers between mother and daughter cells. Examination of hDcp1a and hDcp1b proteins by electrophoresis in mitotic cell extracts showed a pronounced slower migrating band, which was caused by hyper-phosphorylation of the protein. We found that hDcp1a is a phospho-protein during interphase that becomes hyper-phosphorylated in mitotic cells. Using truncations of hDcp1a we localized the region important for hyper-phosphorylation to the center of the protein. Mutational analysis demonstrated the importance of serine 315 in the hyper-phosphorylation process, while other serine residues tested had a minor affect. Live-cell imaging demonstrated that serine mutations in other regions of the protein affected the dynamics of hDcp1a association with the PB structure. Our work demonstrates the control of PB dynamics during the cell cycle via phosphorylation.

## Introduction

Processing bodies (P bodies, PBs) are cytoplasmic structures involved in mRNA decay and mRNA storage. PB structures are detected in all eukaryotes and many of their components are conserved from yeast cells to mammals. PB numbers and size are quite dynamic. Mammalian cells harbor between 3–9 distinct PBs, although these numbers can vary. PBs are 100–300 nms in diameter and are composed of aggregates of electron dense fibrils as observed by electron microscopy. They are readily detectable when cytoplasmic mRNA levels are elevated, and tend to disassemble when mRNA levels drop [1], [2].

PB detection is based mainly on their protein components, such as the decapping enzyme Dcp2 and the exonuclease Xrn1, hinting to their possible role in 5′→3′ mRNA degradation pathways [3]–[5]. Still, even when dispersed in the cytoplasm, PB enzymes do not lose their ability to function in mRNA decay [2]. The 5′ cap structure of mRNA is removed by Dcp2, an enzyme that requires interaction with other proteins for full functionality. In yeast, the Dcp1p protein is a requisite for Dcp2 function [6]–[8], while in human cells additional proteins are required for the Dcp1-Dcp2 interaction [9], [10]. The C-terminus of Dcp1 is a trimerization domain and is required for the decapping activity of the decapping complex [11]. Human cells carry two hDcp1 homologues, hDcp1a and hDcp1b, encoded by two separate genes. The functional difference between the two is unknown and most studies have used the hDcp1a variant as a PB marker.

PB structures are mRNA-protein complexes that are not membrane surrounded. Photobleaching experiments used for measuring protein dynamics in living cells have demonstrated that most PB components exhibit a continuous flux between the cytoplasm and the PB. Uniquely, hDcp2 in PBs shows very low recovery rates after photobleaching indicating that it is a core PB protein [12], in comparison to proteins like hDcp1a that are continuously exchanging with the cytoplasmic pool. While a variety of conditions affect PB formation and disassembly, for instance cell cycle stage [13], cell proliferation rates, nutrient availability and translational stress, the signals that control PB assembly and disassembly are not well understood.

In a previous study we quantified PB mobility in living human cells and demonstrated PB interactions with the microtubule network [12]. This association has been observed in yeast [14] and neuronal cells [15]. We showed that PBs disassembled when transcription and translation were inhibited. Additionally, we found that the disruption of the microtubule network caused an opposite effect of PB assembly [16]. In this study we focused our attention on the time-frame of cell division during which the transcription and translation processes are inhibited, together with microtubule network disassembly. Using live-cell microscopy we demonstrate an increase in PB numbers during S phase, the disappearance of PB structures before mitosis, and their reassembly during cytokinesis. We further analyze the phosphorylation modifications occurring on hDcp1a at the time of cell division.

## Results

### PB disassembly and assembly during cell division

**Figure 1:**
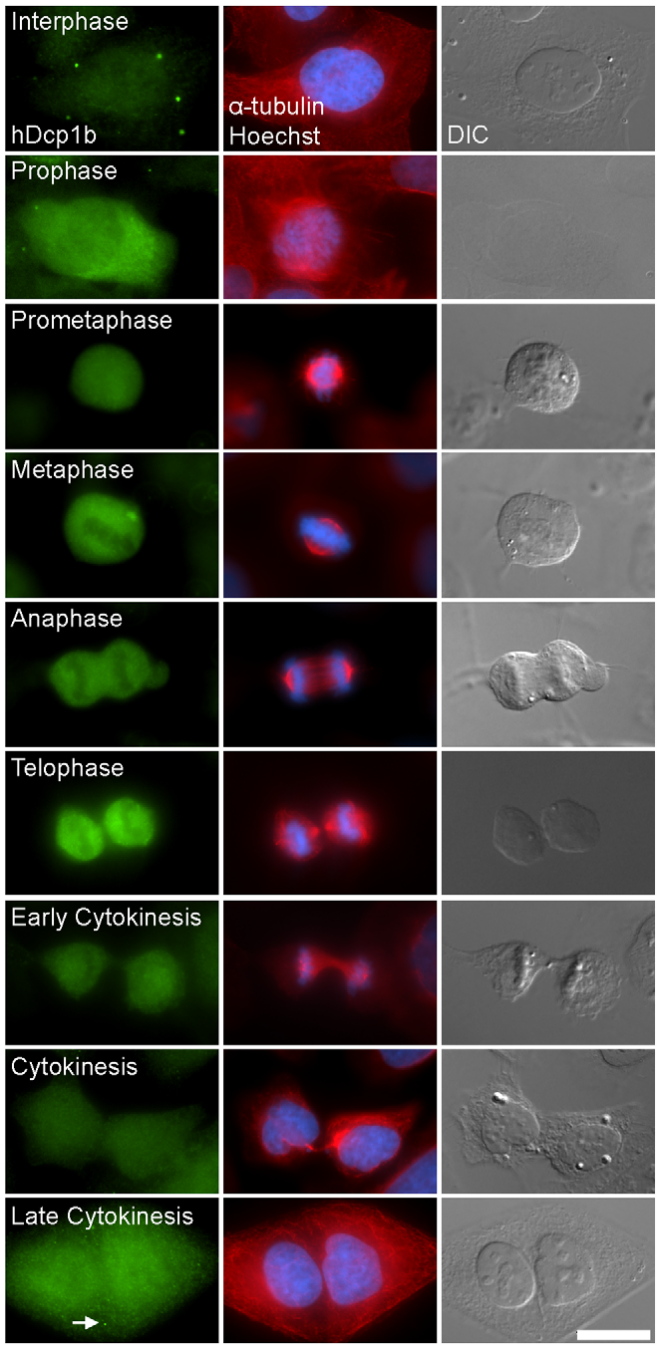
PB assembly and disassembly during the cell cycle. Immunofluoresence staining of endogenous hDcp1b (green), α-tubulin (red), DNA (Hoechst, blue) and DIC images show that PB structures disassemble during cell division. (Bar 20 µm).

**Figure 2:**
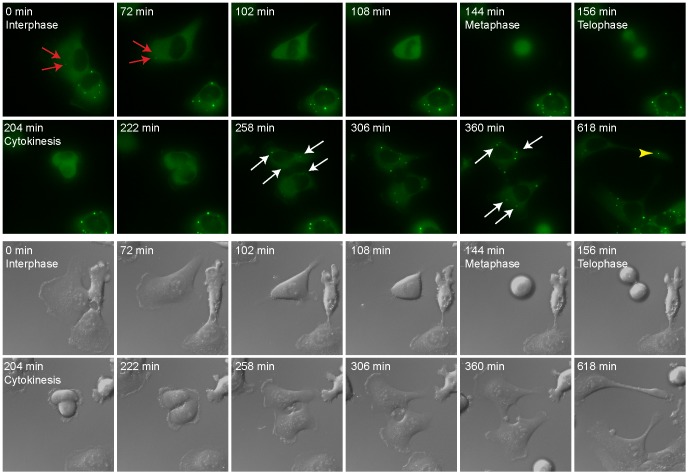
PB assembly and disassembly during cell division in living cells. Cells stably expressing GFP-Dcp1a were simultaneously imaged in GFP and DIC showing the assembly and disassembly of PBs from a movie acquired for 14 hours. Cells were imaged every 6 min. Red arrows: PBs in the cell before mitosis. White arrows: PBs in daughter cells after mitosis. Yellow arrow head: PB in a retraction fiber.

We examined the fate of PBs during cell division. Previous studies, in which PBs/GWBs were marked with an antibody to GW182, found that PBs disassembled upon entry to mitosis [13]. Using antibodies to endogenous hDcp1b (Figure 1) and other PB markers (hDcp1a, hDcp2, Hedls; data not shown) we found that all antigens showed the same behavior and dispersed throughout the cytoplasm of human U2OS cells during mitosis, indicating that the entire PB structure disassembles. The same phenomenon was observed in long-term live cell imaging of a GFP-Dcp1a U2OS stable cell line that allowed the visualization of PB dynamics throughout the cell cycle. Most PBs disappeared during or several minutes before nuclear envelope breakdown (Figure 2 and Movie S1). Occasionally, PBs disappeared some minutes after. The reassembly of PBs after mitosis occurred several minutes after nuclear envelope assembly (Figure 2 and Movie S1). Interestingly, PBs were also observed in the retraction regions formed during cell mobility (Top daughter cell, Figure 2 and Movie S1).

**Figure 3:**
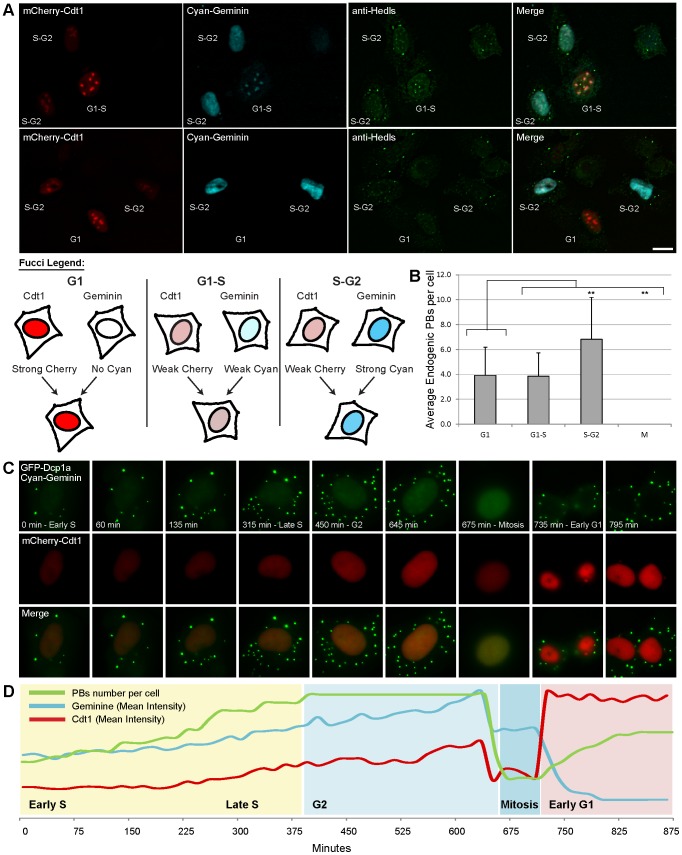
PB numbers increase as cells reach S/G2 phase of the cell cycle. (A) The Fucci markers mCherry-Cdt1 (red) and AmCyan1-Geminin (cyan) were expressed in U2OS cells and then cells were stained with an anti-Hedls (green) antibody to mark PBs. (Bar 20 µm). It was possible to detect the cell cycle phase using the intensity combination of the red and cyan markers in the cell, as explained in scheme below. (B) The number of PBs in each cell was counted and assigned a cell cycle phase according to the Fucci colors. The plot designates the average PB number in each phase (G1 n = 40, G1/S n = 15, S/G2 n = 40, M n = 10). Error bars represent STDEV and a T-Test was performed. (C) U2OS cells stably expressing GFP-Dcp1a were co-transfected with AmCyan1-Geminin and mCherry-Cdt1 and imaged for 15 hours. Frames show the cytoplasmic GFP-Dcp1a signal together with nuclear AmCyan1-Geminin staining that looks green due to the filter used. The plot represents the relative intensity analysis of all markers as quantified throughout the movie. Red – mean Cdt1 intensity, cyan – mean Geminin intensity, green – number of PBs.

We did not observe a correlation between the number of PBs present within the cell before and after mitosis. For instance, in Figure 2 there were two detectable PBs in the pre-mitotic cell, while after mitosis the two daughter cells contained 4 or 7 PBs, respectively. To carefully examine PB numbers during the cell cycle we made use of the Fucci system that allows the identification of cells in various phases of the cell cycle using two fluorescent cell cycle markers. The two Fucci vectors encode for Cdt1 or Geminin, proteins whose levels fluctuate differentially throughout the cell cycle. Cdt1 levels peak in G1 phase, and then as cells transition into S phase, Cdt1 levels fall and Geminin levels rise, remaining high until the cells are back in G1. Cells control Cdt1 and Geminin levels post-translationally using ubiquitination to target the unwanted proteins for proteasomal degradation. We expressed a combination of mCherry-Cdt1 and AmCyan1-Geminin in U2OS cells and labeled PBs using an anti-Hedls antibody (Figure 3A). Counting PBs at different steps of the cell cycle showed that PB numbers increased from an average of 4±2 per cell during G1 and G1/S to an average of 7±3 in S/G2, and were not detectable during mitosis (Figure 3B). The gradual change in PB numbers through the cell cycle could also be followed in living cells expressing GFP-Dcp1a and the Fucci markers (Figure 3C,D and Movie S2). An increase in PB numbers was observed during late S, which then remained steady until cell division. Finally, the daughter cells contained less PBs than the mother cell prior to mitosis (Figure 3C,D and Movie S2). Altogether, we find that visible PB structures increase in number as the cell approaches S phase, and then finally disassemble during mitosis during which PB components are dispersed in the dividing cell. PB structures reform immediately after daughter cell formation.

### Dcp1a protein is hyper-phosphorylated during mitosis

**Figure 4:**
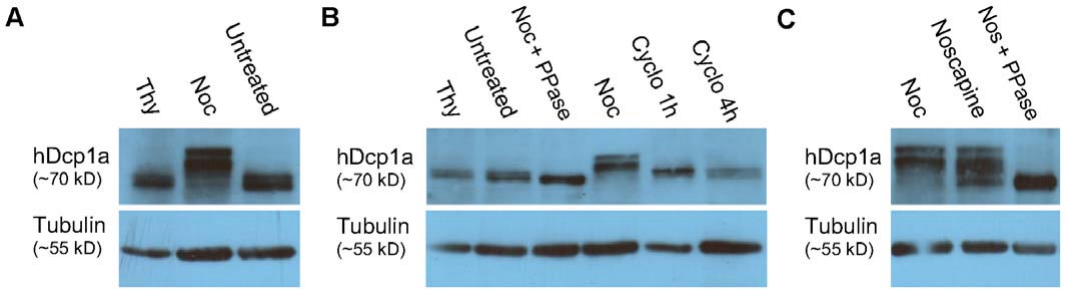
Dcp1a is hyper-phosphorylated during cell division. Western blot analysis of (A) endogenous hDcp1a protein in U2OS cell extracts during interphase (untreated), metaphase (nocodazole block, Noc) and at G1/S (thymidine block, Thy), showed the appearance of slower migrating Dcp1a bands in metaphase cells. (B) Treatment of U2OS protein extracts from metaphase cells with a phosphatase (Noc+PPase) caused a reduction in the molecular weight of hDcp1a, compared to untreated, G1/S blocked (Thy), and metaphase blocked cells (Noc). This demonstrated that Dcp1a is hyper-phosphorylated during mitosis. Treatment with cycloheximide (Cyclo) for 1 or 4 hrs did not change the mobility of hDcp1a indicating that hyper-phosporylation is cell cycle dependent. (C) Shift in mobility due to hyper-phosphorylation in mitotic cells is seen using two different cell cycle blockers, nocodazole (Noc) and noscapine. Similarly, phosphatase treatment (Nos+PPase) caused a reduction in the molecular weight of Dcp1a from noscapine treated cells. Tubulin was used as a loading control.

It is known that phosphorylation regulates the assembly/disassembly of structures during mitosis [17]. We next examined whether post-translational modifications are occurring on PB components during PB disassembly, focusing on the hDcp1a protein. Western blots to endogenous hDcp1a showed that changes in PB integrity during the cell cycle did not involve a reduction in protein levels. Instead, slower band migration was observed for the endogenous hDcp1a protein coming from protein extracts of synchronized mitotic cells (nocodazole block), indicating the occurrence of post-translational modifications on the protein during mitosis (Figure 4A). Several slow migrating hDcp1a bands were observed in mitotic cells, while cells synchronized to G1/S (thymidine block) showed a faster migrating doublet similar to untreated cells (Figure 4A). The same mobility patterns were observed in HeLa cells (Figure S1A). Slower migrating bands were not observed in cells treated with nocodazole for only 30 minutes (not shown), a treatment that disassembles microtubules [12], nor with cycloheximide for up to 4 hours (Figure 4B and S1A), a treatment that inhibits translation.

Treatment of protein extracts from mitotic cells with a phosphatase led to higher mobility of hDcp1a and to the disappearance of the slower migrating bands (Figure 4B and S1A). Moreover, the band from the phosphatase-treated extract did not present as a doublet as seen in the extracts from untreated cells, and had even higher mobility than the hDcp1a protein bands from the control and G1/S blocked cells. The hyper-phosphorylation of Dcp1a was also observed when synchronizing cells by the means of noscapine, which interferes with microtubule function and thus with cell division (Figure 4C). Altogether, this means that during interphase hDcp1a is a phospho-protein, that later undergoes hyper-phosphorylation prior to or during mitosis. Hyper-phosphorylation was detected also for GFP-Dcp1a and GFP-Dcp1b (Figure S1B).

### Amino acids situated within the 200–380 aa region of the Dcp1a protein are phosphorylated during mitosis

**Figure 5:**
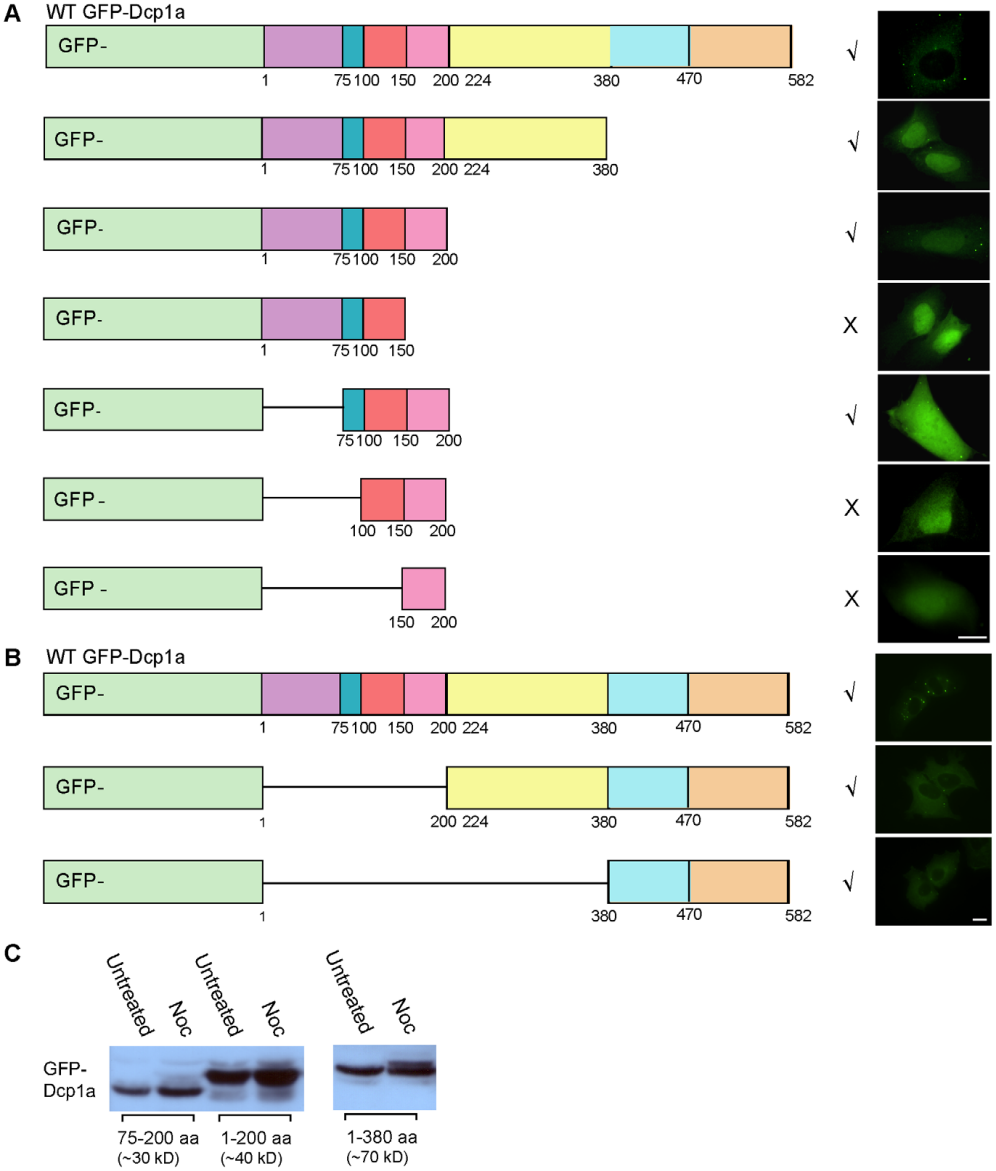
Phosphorylation of Dcp1a occurs in 200–380 region of the protein. GFP constructs containing different fragments of the hDcp1a protein were transfected into U2OS cells and their assembly into PBs was monitored. The symbol √ indicates accumulation in PBs and the symbol×indicates no accumulation. (A) C-terminal truncations showing that region 1–200 is important for assembly into PBs. (B) N-terminal truncations. (Bar 20 µm). (C) Change in SDS-PAGE mobility in extracts from mitotic cells was detected for the 1–380 aa GFP-Dcp1a truncated protein, but not in the 1–200 and 75–200 aa forms. The blots were reacted with anti-GFP. Tubulin was used as a loading control.

To identify the region in hDcp1a undergoing hyper-phosphorylation during mitosis we first generated a series of GFP-Dcp1a truncated constructs containing different regions of the protein. We first determined which is the smallest domain of Dcp1a that still assembles in PBs. Figure 5A shows that hDcp1a truncated of its mid- and C-terminal regions (protein containing only aa's 1–200) could assemble in PBs while the shorter N-terminal 1–150 aa protein did not assemble in PBs. All the truncated proteins were tested and did not have a dominant negative effect on the formation of endogenous PBs in the transfected cells.

The N-terminal 1–133 region in hDcp1a is similar to the N-terminal regions of hDcp1b, and to two regions of the *S. cerevisiae* Dcp1p protein homologue [18]. This region is important for decapping activity, since site-specific mutations in two residues of hDcp1a (D20A and R59A) reduced the decapping activity of immunoprecipitated complexes [18]. We therefore generated additional constructs based on the smallest assembling truncated protein (1–200) in which the regions with the critical amino acids (aa's 20 and 59) were removed. Truncated versions containing the region 75–200 of hDcp1a assembled in PBs indicating that these specific N-terminal residues (in region 1–75) were not necessary for assembly into PBs. However, further dissection of this region showed that truncated proteins containing amino acids 150–200 or 100–200 were not able to assemble into PBs. To identify the region of hDcp1a that undergoes hyper-phosphorylation we expressed these truncated forms of GFP-Dcp1a that assemble in PBs, performed a nocodazole block and collected mitotic cells. The N-terminal portion of Dcp1a (75–200 or the 1–200 regions) did not show a mobility shift in Western blots (Figure 5C), meaning that this probably was not the region responsible for the hyper-phosphorylation.

Next we generated N-terminally truncated forms of hDcp1a (200–582 or 380–582) lacking the 75–200 region. These were still able to assemble into PBs (Figure 5B), implying that it is not necessarily a contiguous region in the protein that is responsible for hDcp1a targeting to PBs. Finally, hyper-phosphorylation in mitotic cells was detected for the truncated 1–380 form lacking the C-terminus (Figure 5C). We therefore concluded that the region 200–380 contains amino acid residues that are hyper-phosphorylated during mitosis.

### Serine 315 is involved in Dcp1a hyper-phosphorylation during mitosis

The above mentioned 200–380 region of hDcp1a contains a high number of putative phosphorylations sites (21 serines, 14 threonines and 1 tyrosine) (Figure S2). A previous study provided a scored list of serine and threonine residues that are prone to phosphorylation during mitosis [19]. We chose to focus on serines in positions 315 and 319 (found within the 200–380 region of hDcp1a), which received high phosphorylation scoring in this analysis. As a control we chose serines 522 and 523 from the C-terminus, which were also highly scored but according to the truncations are probably not phosphorylated during mitosis (Figure S2).

**Figure 6:**
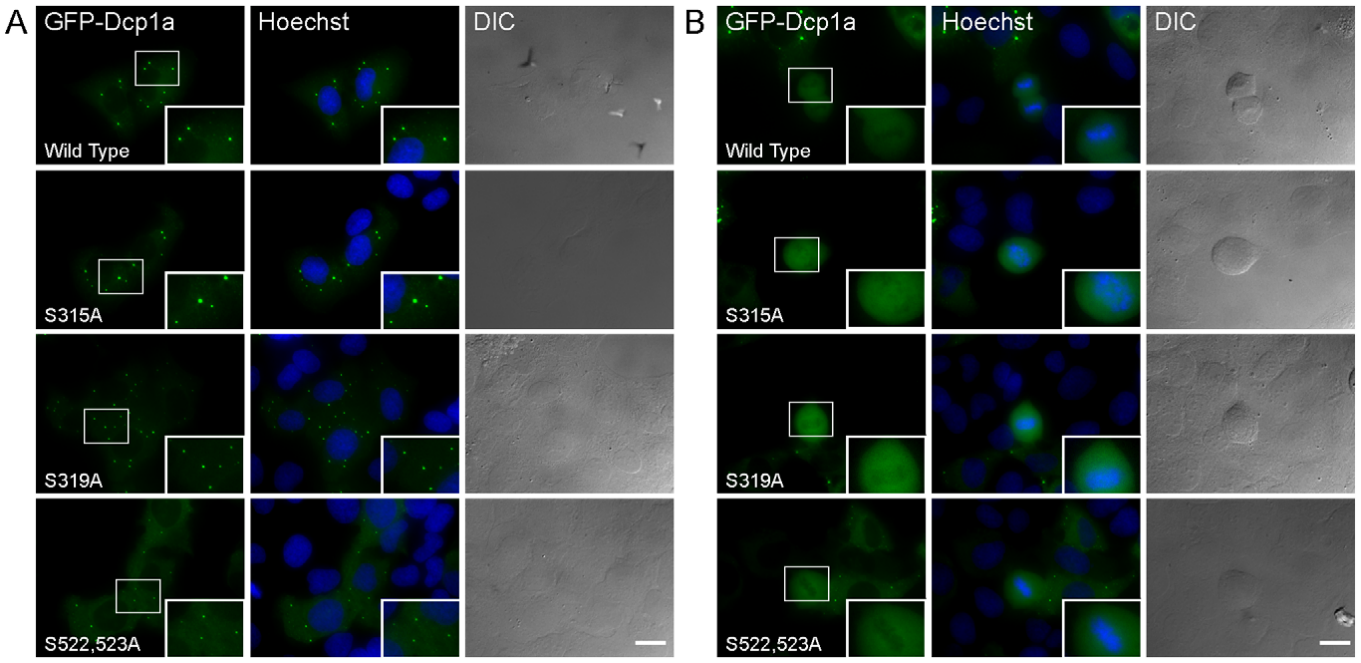
Serine mutated Dcp1a proteins assembled into PBs. (A) Serine to alanine mutated GFP-Dcp1a proteins assembled in PBs in U2OS cells, and (B) disassembled during mitosis. Enlarged insets are boxed. DNA was counterstained with Hoechst. (Bar 20 µm).

**Figure 7:**
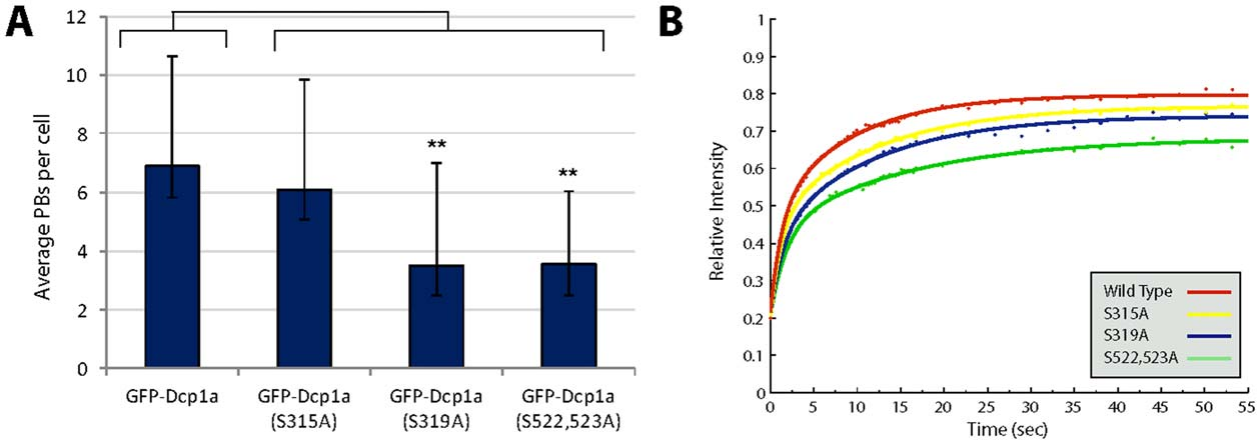
Serine 522 and 523 mutations affect Dcp1a association/dissociation dynamics in PBs. (A) Plot showing the average number of PBs in cells expressing the different serine mutated forms of Dcp1a. A statistically significant reduction in PB numbers was seen in cells expressing the S522,523A and S319A mutations (n = 40). Error bars represent STDEV and a T-Test was performed. (B) PBs containing the different serine mutated proteins were photobleached and the kinetics of recovery were analyzed. The curves represent an average of 20 PBs in 10 cells. The recovery curves were statistically different as seen by Mann-Whitney (non parametric test). The recovery curves were fit by Matlab and the t_1/2_ recovery times and fixed fractions were calculated and showed a reduction in Dcp1a association with the PB structure in cells expressing the S522,423A mutant.

Using site specific mutagenesis we mutated all 4 serines to alanines in the GFP-Dcp1a construct, and generated the following 4 constructs: GFP-Dcp1a S315A, S319A, S315+319A, S522+523A. All mutated plasmids were then transiently transfected into U2OS cells. In all cells the expressed mutated hDcp1a proteins were found in PBs (Figure 6A), and disassembled during mitosis (Figure 6B). We stably integrated the wild type and mutated plasmids into U2OS cells. Counting of PBs in the cell lines showed a statistically significant reduction in PB numbers in the cells expressing GFP-Dcp1a S319A and S522+523A (Figure 7A). This could indicate that these mutated forms were not able to correctly assemble in PBs. Therefore, we next examined whether there was a change in the dynamics of the hDcp1a protein during association and dissociation with PB structures, due to these mutations. The association/dissociation rates of PB components are measured using the fluorescence recovery after photobleaching (FRAP) method [12]. FRAP recovery rates demonstrated that indeed the S522,523A mutations caused a significantly faster interchange of the hDcp1a protein within the PBs (t_1/2_ of recovery = 3.3 sec for wild type hDcp1a, and 2.28 sec for S522,523A) indicating a problem in association with the PB structure (Figure 7B). Moreover, there was a change in the immobile Dcp1a fraction within the PB, which was typically 32% in cells expressing wild type hDcp1a, and was reduced to 20% with the S522,523A mutant, once again indicating an assembly defect for this mutant. However, the S315A and S319A mutations did not significantly change the dynamics (Figure 7B).

**Figure 8:**
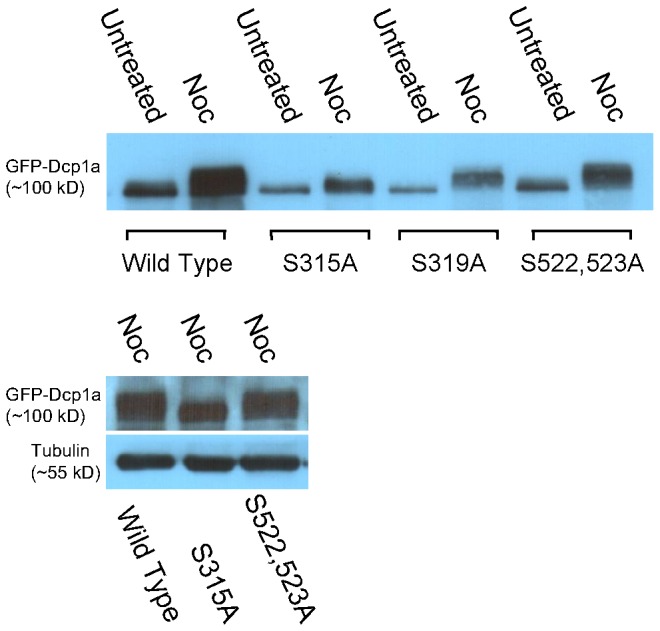
Serine 315 is important for the hyper-phosphorylation of Dcp1a during cell division. Top - The S319A and S522,523A mutated GFP-Dcp1a proteins showed prominent hyper-phosphorylation patterns compared to the S315A protein. Bottom - blot comparing the mobility shifts of the mutated proteins from mitotic cell extracts showing that S315A is the least affected.

We then examined whether the hyper-phosphorylation occurring on GFP-Dcp1a during mitosis as seen by a mobility shift in Western blots, was affected by the serine mutations in hDcp1a. Figure 8A shows that hDcp1a hyper-phosphorylation in mitotic cells was highly reduced in the S315A mutant (Figure 8A and S3A,B), although the endogenous hDcp1a protein was hyper-phosphorylated as usual (Figure S3A). In contrast, the S319A and the S522,523A mutants continued to show hyper-phosphorylated bands in the mitotic cell extracts (Figure 8A and S3A,B), although the mobility shift was less pronounced in S319A. Altogether, these results demonstrate that serine residue S315 is a key player in the hyper-phosphorylation that Dcp1a undergoes during mitosis.

## Discussion

The Dcp1-Dcp2 interaction is required for 5′ mRNA decapping from yeast to metazoa. In humans this interaction occurs in multimeric decapping complexes that require enhancers of decapping 3 and 4 (EDC3, EDC4), and the DEAD-box RNA helicase DDX6. A structural study showed that Dcp1a proteins can form trimers via a C-terminal trimerization domain and can also heterodimerize with Dcp1b [11]. Dcp1a can interact with Dcp2 and EDC4 independently of its interaction with EDC3 and DDX6. PB size can change in response to a variety of cell cycle and metabolic signals, and this may depend on the oligomerization capabilities of some of its components such as hDcp1a [5], [12], [13], [20]. Indeed, the oligomerization traits of hDcp1a can lead to the formation of very large PBs observed when fluorescently tagged hDcp1a is highly overexpressed in human cells [20]. hDcp1a dynamic properties in PBs as measured by FRAP were dependent on PB size, and the comparison of FRAP recoveries of different PB proteins has suggested that hDcp1a shuttles in and out of PBs independently of the RNA substrates [20].

While studying PB dynamics in living cells we found that we could follow PB assembly and disassembly during the cell cycle. The numbers of detectable PBs increases as cells proceed through S phase, and remain high until cell division. PBs disassemble during mitosis and reassemble immediately when interphase resumes. There are less PBs in daughter cells compared to the number of PBs in the mother cell before division. Since phosphorylation is a major regulator of structural integrity in cells during mitosis we decided to examine the phosphorylation profile of hDcp1a during mitosis. We found that hDcp1a and hDcp1b proteins undergo hyper-phosphorylation during mitosis. Several findings have indicated that hDcp1a is a phospho-protein under normal conditions. The yeast Dcp1 protein was found to migrate as two bands in SDS-PAGE experiments and was later shown to be a phospho-protein [21], [22]. Similar results were observed in a variety of mammalian cell lines [23]. Interestingly, the relative intensity of these differentially migrating bands changed during mouse brain development or following differentiation of P19 neuronal cells, culminating with only one hDcp1a band. These data demonstrated that hDcp1a phosphorylation levels can be reduced during differentiation. This study also examined a number of serine and threonine residues and found that mutation of S142, S144, S319, T321 or S353, had no effect on the mobility of YFP-Dcp1a. However, when mutated at S315 the hDcp1a protein migrated as a single band, and when a triple mutation S315/S319/T321 was expressed, the migration was even slower. Following arsenate treatment hDcp1a became hyper-phosphorylated but the change in mobility was not affected by separately mutating S315, S319 or T321, whereas the triple S315/S319/T321 mutant did not show a mobility shift [23]. Altogether, these data demonstrate that hDcp1a is a phospho-protein under normal conditions, and can be hyper-phosphorylated following arsenate treatment.

Phosphatase treatment of the hyper-phosphorylated form of hDcp1a from mitotic cells showed that the protein in interphase is already a phospho-protein and accumulates additional phospho-modifications during mitosis. The region important for hDcp1a hyper-phosphorylation during mitosis was identified within the center of the protein (aa's 200–380). We chose to mutate two serine amino acids within this region (315 and 319) and two serine residues from the C-terminus (522 and 523). These amino acid positions were shown to be highly prone to phosphorylation by a proteomic screen [19]. Serine 315 mutation had a dramatic effect, while the others did not. During mitosis, very little hyper-phosphorylation was detected on the S315A mutated protein. Since the hyper-phosphorylation occurs probably on several hDcp1a residues as deduced from the significant mobility shifts, it would suggest that phosphorylation on serine 315 is critical for initiation of this process during mitosis. On the other hand, even though stably overexpressed, this mutated protein did not have a negative effect on PB integrity and could in fact assemble in PBs.

The mutations in the C-terminus of hDcp1a (residues 522 and 523) were not important for the hyper-phosphorylation of the protein during mitosis. However, these amino acid substitutions changed the association rates of hDcp1a with the PBs as measured by FRAP, indicating that this region plays an important role in association with the PB structure. Indeed, the C-terminal portion of hDcp1a has been shown to be an essential region for the trimerization of hDcp1a, in which it folds into three kinked α-helices, and is also important for localization to PBs [11].

Under endogenous conditions of cell division, when cells divide and cease to transcribe in conjunction with the loss of the MT network, PBs disassemble. We found that Dcp proteins are hyper-phosphorylated during cell division, in particular hDcp1a. On the other hand, drug treatments such as nocodazole or cycloheximide that increase PB abundance, did not affect the phosphorylation status of the protein. Serine 315 of hDcp1a is important for hDcp1a phosphorylation during translational stress such as arsenate treatment [23] or sorbitol or anisomycin [24]. The latter study showed that S315 is phosphorylated by the JNK kinase in response to IL-1 treatment, and that hDcp1a phosphorylation during stress is associated with PB dispersal. Interestingly, decapping activity of S315 mutant hDcp1a was not abolished.

Other PB proteins are phospho-proteins, such as GW182 [25], Pat1 in yeast [26], and Dcp2 in yeast [27]. *S. cerevisiae* Dcp2 is phosphorylated by the Ste20 kinase during a variety of stresses such as glucose deprivation, oxidative stress, and high cell density, and in this case the phosphorylation is required for the localization in PBs [27]. Pat1 in yeast is phosphorylated by PKA, and this disrupts interaction with other PB components such as Dhh1 [26]. Overall, it is possible that PB assembly, disassembly and even cytoskeletal association and transport are controlled by phosphorylation events.

Why then is Dcp1a hyper-phosphorylated during mitosis? This might be a mechanism for protecting mRNAs from degradation during cell division. mRNAs must not be degraded during mitosis otherwise the cell would enter a new cycle with no available mRNAs or proteins. Indeed, most translation shuts down during mitosis [28] probably via mechanisms that regulate initiation [29]. It was suggested that much of the mRNAs are retained in polysomes during mitosis [30]. Also, mRNAs are stabilized when translation inhibitors are added to cells [31]–[33]. It has been hypothesized [34] that mRNA stabilization is a requirement for the cell during cell division (and similarly when using translation inhibitors). This hypothesis postulated that mRNA stabilization during mitosis cannot possibly depend on the binding of thousands of stabilizing molecules to mRNAs, but most likely relies on the inactivation of mRNA degradation factors during mitosis. Our data suggest that PB disassembly during mitosis is regulated by specific phosphorylation events that disrupt the interactions between key PB components, which may lead to inactivation of the decapping machinery. PB dispersal alone does not protect mRNAs from degradation since even under such conditions PB proteins maintain their competence for mRNA decay [2], [35]. However, phosphorylation of yeast Dcp2 had a positive effect on mRNA stability of several mRNAs [27] as did the phosphorylation of hDcp1a on several IL-1 responsive mRNAs [24]. It therefore seems plausible to suggest that disassembly of PBs in conjunction with hyper-phosphorylation of key components such as hDcp1a would render PB proteins inactive during cell division, thus providing a time-frame during which mRNA decay is significantly reduced and to allow for fast recovery of post-mitotic cellular processes.

## Materials and Methods

### Plasmids and site directed mutagenesis

The GFP-Dcp1a was previously described [12]. The truncated forms of GFP-Dcp1a were obtained by the following restriction reactions on GFP-Dcp1a: 1–200: digested with XbaI; 1–150: digested with PstI. For 150–200: GFP-Dcp1a was digested with HindIII and PstI, and the fragment was subcloned into the peGFP-C1 vector. For 75–200: This region was amplified by RT-PCR from GFP-Dcp1a using primers – ATAGAATTCGCACAATCTAGTTGAACCAGGAA, ATACCCGGGTTCCAAATAACTCTTCTACCGT, digested with EcoRI and XbaI and subcloned into peGFP-C1. For 100–200: The amplified 75–224 region was digested with HindIII and XbaI and then subcloned into peGFP-C1.

The previously constructed GFP-Dcp1a plasmid was mutated using the QuikChange II Site-Directed Mutagenesis kit (Stratagene). Serine 315 was mutated to alanine using primers – CTACACAATCCCGTTGGCCTGTTCTCAGTCCC, GGGACTGAGAACAGGGGAACGGGATTGTGTAG. Serine 319 was mutated to alanine using primers - CGTTGAGCCCTGTTCTCGCCCCACTCTGCCAGC, GCTGGCAGAGTGGGAGCCGAACAGGGCTCAACG.

Both serines 315 and 319 were mutated to alanine using primers - GTTGGCCCCTGTTCTCGCCCACTCTGCCAG, CAATAGTTAGAGGAGAAGGGCGGCGGCTTTCCTCTCAA.

Both serines at 522 and 523 were mutated to alanine using primers - CCTTGAGAGGAAAGCCGCGCCCCTTCTCCTCTAACTATT, CAATAGTTAGAGGAGAAGGGCGGCGGCTTTCCTCTCAA.

### Cell culture and transfections

Human U2OS cells were maintained in low glucose DMEM (Biological Industries, Israel) and HeLa cells in high glucose DMEM (Gibco) containing 10% FBS (HyClone). The following U2OS stable lines were generated: GFP-Dcp1a, GFP-Dcp1a S315A, GFP-Dcp1 S319A, GFP-Dcp1a S522,523A. For Western blotting of GFP-Dcp1a truncations, cells were transiently transfected with Lipofectamine (Invitrogen). The Fucci system (Clontech) was used for cell cycle phase detection. For G1 phase detection, pRetroX-G1-Red vector (mCherry-hCdt1) was used, and for S/G2 the Phase pRetroX-SG2M-Cyan vector (AmCyan-hGeminin). Fucci vectors were transiently transfected into U2OS cells with the PolyJet transfection reagent (SignaGem Laboratories).

### Immunofluorescence

Immunofluorescence was performed as previously described [12]. Primary antibodies: rabbit anti-hDcp1a, rabbit anti-hDcp1b, anti-Dcp2 (J. Lykke-Andersen, University of Colorado, Boulder, CO), mouse anti-hDcp1a (Abnova), mouse anti-Hedles (Santa Cruz), rabbit anti-α-tubulin (Abcam). Secondary Abs: anti-rabbit and anti-mouse Cy3 (Jackson ImmouResearch). Nuclei were counterstained with Hoechst 33342 and coverslips were mounted in mounting medium.

### Cell synchronization

For the various treatments cells were treated with: 600 nM nocodazole, 5 µg/ml cycloheximide (Sigma). For cell cycle synchronization, cells were arrested at G1/S using a thymidine block. Briefly, cells were cultured for 1 day and then incubated in medium containing 5 mM thymidine (Sigma) for U2OS cells and 2 mM thymidine for HeLa cells for 24 hours. For a mitotic block, cells were incubated with medium containing 600 nM nocodazole or 25 µM noscapine for 16 hrs. Mitotic cells were washed off the plates. Cells were then washed briefly and cell extracts were prepared for Western blotting.

### Western blotting

SDS-PAGE and Western blotting were performed as previously described [12]. When appropriate, extracts were further treated with 400 u of lambda protein phosphatase (New England Biolabs) for 1 hr at 30°C. Primary antibodies used were mouse anti-hDcp1a (Abnova), rabbit anti-hDcp1a, (J. Lykke-Andersen), mouse anti-α-tubulin (Abcam), mouse anti-GFP (Covance), mouse anti-GFP (Roche). The secondary antibody was a HRP-conjugated goat anti-rabbit or anti-mouse IgG (Sigma). Immunoreactive bands were detected by the Enhanced Chemiluminescence kit (ECL, Pierce).

### Fluorescence microscopy, live-cell imaging and data analysis

Wide-field fluorescence images were obtained using the Cell∧R system based on an Olympus IX81 fully motorized inverted microscope (60× PlanApo objective, 1.42 NA) fitted with an Orca-AG CCD camera (Hamamatsu), rapid wavelength switching, and driven by the Cell∧R software. For time-lapse imaging, cells were plated on glass-bottomed tissue culture plates (MatTek, Ashland, MA) in medium containing 10% FCS at 37°C. The microscope is equipped with an enclosure incubator which includes temperature and CO2 control (Life Imaging Services, Reinach, Switzerland). For long-term imaging of the cell cycle, several cell positions were chosen and recorded by a motorized stage (Scan IM, Märzhäuser, Wetzlar-Steindorf, Germany). In these experiments, cells were typically imaged in 3D (4 Z planes per time point) every 6 minutes, at 3.33 µm steps (Figure 2) or every 15 minutes at 2 µm steps (Figure 3). For presentation of the movies, the 4D image sequences were transformed into a time sequence using the maximum projection option in the Cell∧R software.

### FRAP

FRAP experiments were performed using a 3D-FRAP system (Photometrics) built on an Olympus IX81 microscope (636 Plan-Apo, 1.4 NA) equipped with an EM-CCD (Quant-EM, Roper), 491 nm laser, Lambda DG-4 light source (Sutter), XY&Z stages (Prior), and driven by MetaMorph (Molecular Devices). Experiments were performed at 37°C with 5% CO2 using a live cell chamber system (Tokai). For each acquisition, PBs were bleached using the 491 nm laser. Six pre-bleach images were acquired. Post-bleach images were acquired with a sequence of 2 time frequencies: 57 images every 350 msec and 12 images every 3 sec. The experiments were analyzed using ImageJ macros previously described [12]. Data from at least 10 experiments for each cell line were collected and the averaged FRAP measurements were fitted by Matlab.

## Supporting Information

Movie S1 **PB assembly and disassembly during cell division in living cells.** Cells stably expressing GFP-Dcp1a were simultaneously imaged in GFP and DIC showing the assembly and disassembly of PBs during a movie of 14 hours. Cells were imaged every 6 min.
(MOV)

Movie S2 **PB numbers during the cell cycle.** U2OS cells stable for GFP-Dcp1a were co-transfected with AmCyan1-Geminin and mCherry-Cdt1 and imaged for 15 hours. The movie shows the cytoplasmic GFP-Dcp1a signal together with nuclear AmCyan1-Geminin staining (looks green due to the filter used). The increase in PBs during S can be seen for the marked cell and also for the unlabeled cell above, which also undergoes mitosis at a similar time. Cells were imaged every 15 min.
(MOV)

Figure S1 **Hyper-phosphorylation of Dcp1a during mitosis.** (A) Treatment of U2OS or HeLa protein extracts before SDS-PAGE with a phosphatase (Noc+PPase) caused a reduction in the molecular weight of hDcp1a, compared to untreated, G1/S blocked (Thy), and metaphase blocked cells (Noc), and the appearance of slower migrating Dcp1a bands. This demonstrated that Dcp1a is hyper-phosphorylated during mitosis. Treatment with cycloheximide (Cyclo) for 1 or 4 hrs did not change the mobility of hDcp1a indicating that hyper-phosporylation is cell cycle dependent. (B) Shift in mobility due to hyper-phosphorylation in mitotic cells is seen also for GFP-Dcp1a and GFP-Dcp1b using an anti-GFP antibody. Phosphatase (Noc+PPase) treatment caused a reduction in the molecular weight of GFP-Dcp1a and Dcp1b, compared to control. Tubulin was used as a loading control.
(TIF)

Figure S2 **Putative phosphorylation sites in the hDcp1a protein.** The central region of Dcp1a is marked in red (200–380, as used in figure 5). Serine, threonine, and tyrosine residues are marked in green. Mutated amino acids are marked in yellow.
(TIF)

Figure S3 **Mutation S315A reduces hyper-phosphorylation of GFP-Dcp1a.** (A) No hyper-phosphorylation of hDcp1a S315A mutated protein was observed in mitotic cells (Noc) expressing GFP- hDcp1a S315A (100 kD) compared to the endogenous Dcp1a protein (70 kD) which did show hyper-phosphorylated Dcp1a bands. The blot was reacted with anti-Dcp1a. (B) The S319A and S522,523A mutated GFP-Dcp1a proteins showed prominent hyper-phosphorylation patterns compared to the S315A protein. Tubulin was used as a loading control.
(TIF)
