# Supplementary figures and images for: The P Body Protein Dcp1a Is Hyper-phosphorylated during Mitosis
Source: PLoS One. 2013 Jan 2;8(1):e49783. doi: 10.1371/journal.pone.0049783 (PMC3534667; doi:10.1371/journal.pone.0049783)

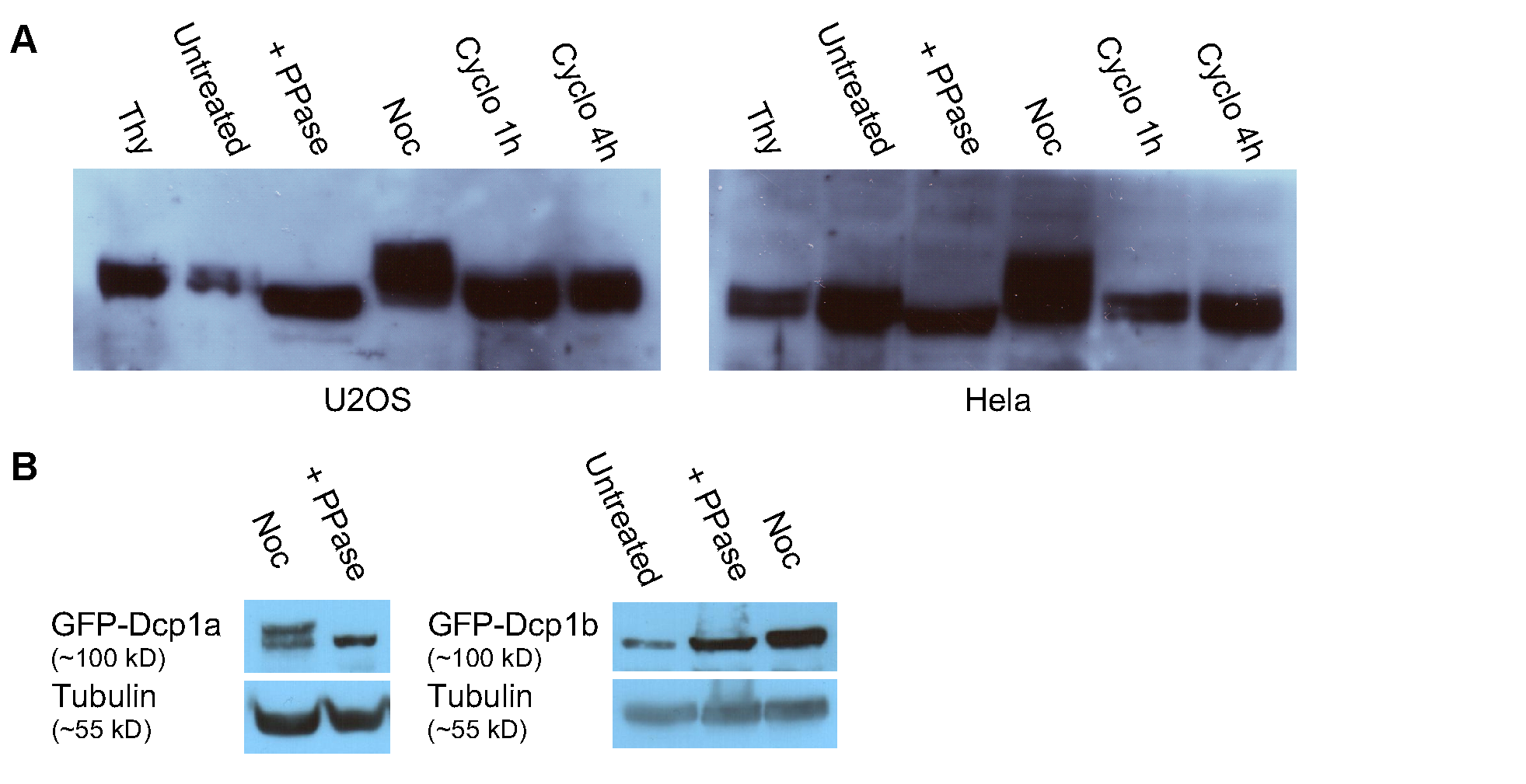

Supplement: Figure S1 — Hyper-phosphorylation of Dcp1a during mitosis. (A) Treatment of U2OS or HeLa protein extracts before SDS-PAGE with a phosphatase (Noc+PPase) caused a reduction in the molecular weight of hDcp1a, compared to untreated, G1/S blocked (Thy), and metaphase blocked cells (Noc), and the appearance of slower migrating Dcp1a bands. This demonstrated that Dcp1a is hyper-phosphorylated during mitosis. Treatment with cycloheximide (Cyclo) for 1 or 4 hrs did not change the mobility of hDcp1a indicating that hyper-phosporylation is cell cycle dependent. (B) Shift in mobility due to hyper-phosphorylation in mitotic cells is seen also for GFP-Dcp1a and GFP-Dcp1b using an anti-GFP antibody. Phosphatase (Noc+PPase) treatment caused a reduction in the molecular weight of GFP-Dcp1a and Dcp1b, compared to control. Tubulin was used as a loading control. (TIF) [file pone.0049783.s003.tif]

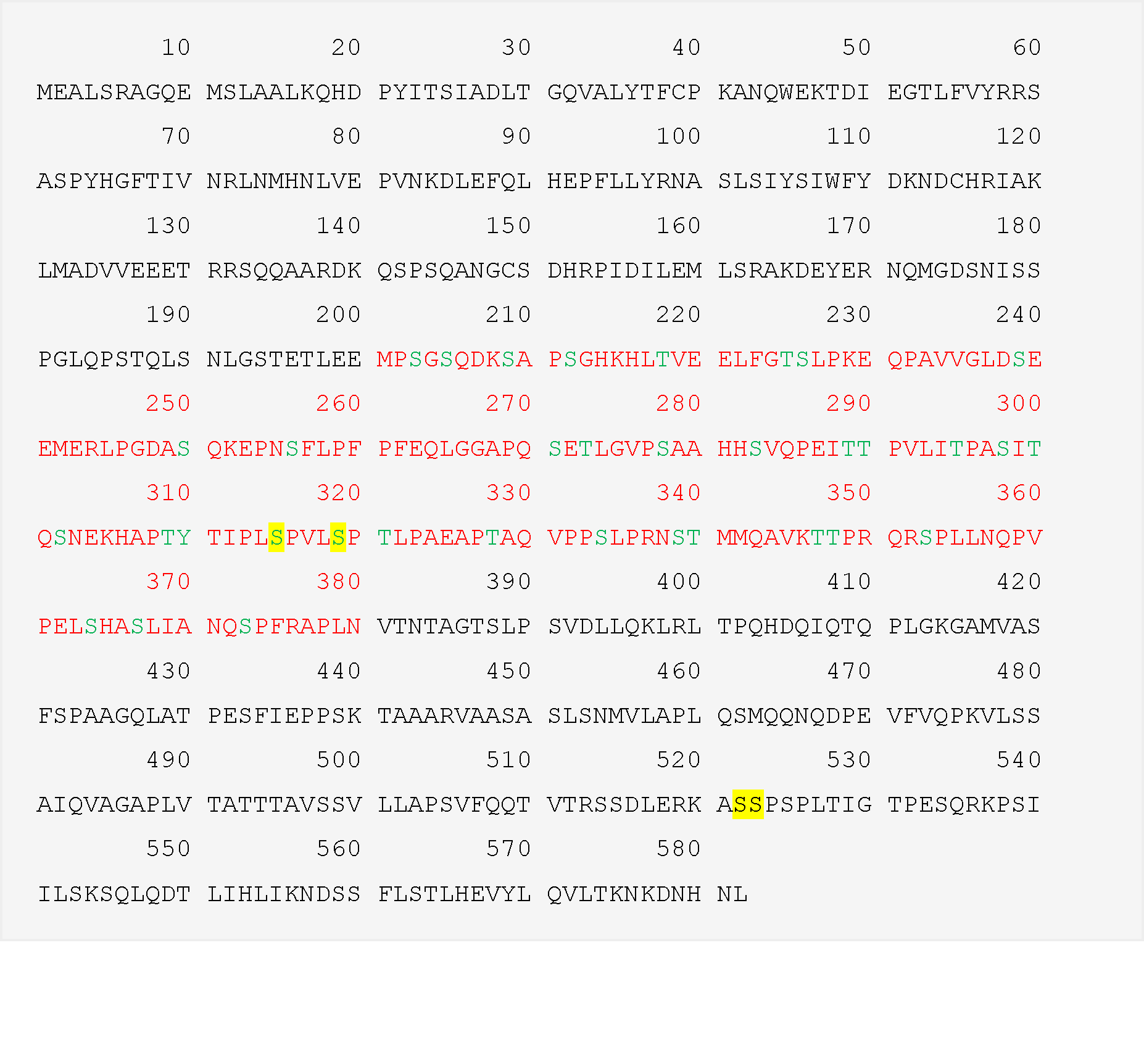

Supplement: Figure S2 — Putative phosphorylation sites in the hDcp1a protein. The central region of Dcp1a is marked in red (200–380, as used in figure 5). Serine, threonine, and tyrosine residues are marked in green. Mutated amino acids are marked in yellow. (TIF) [file pone.0049783.s004.tif]

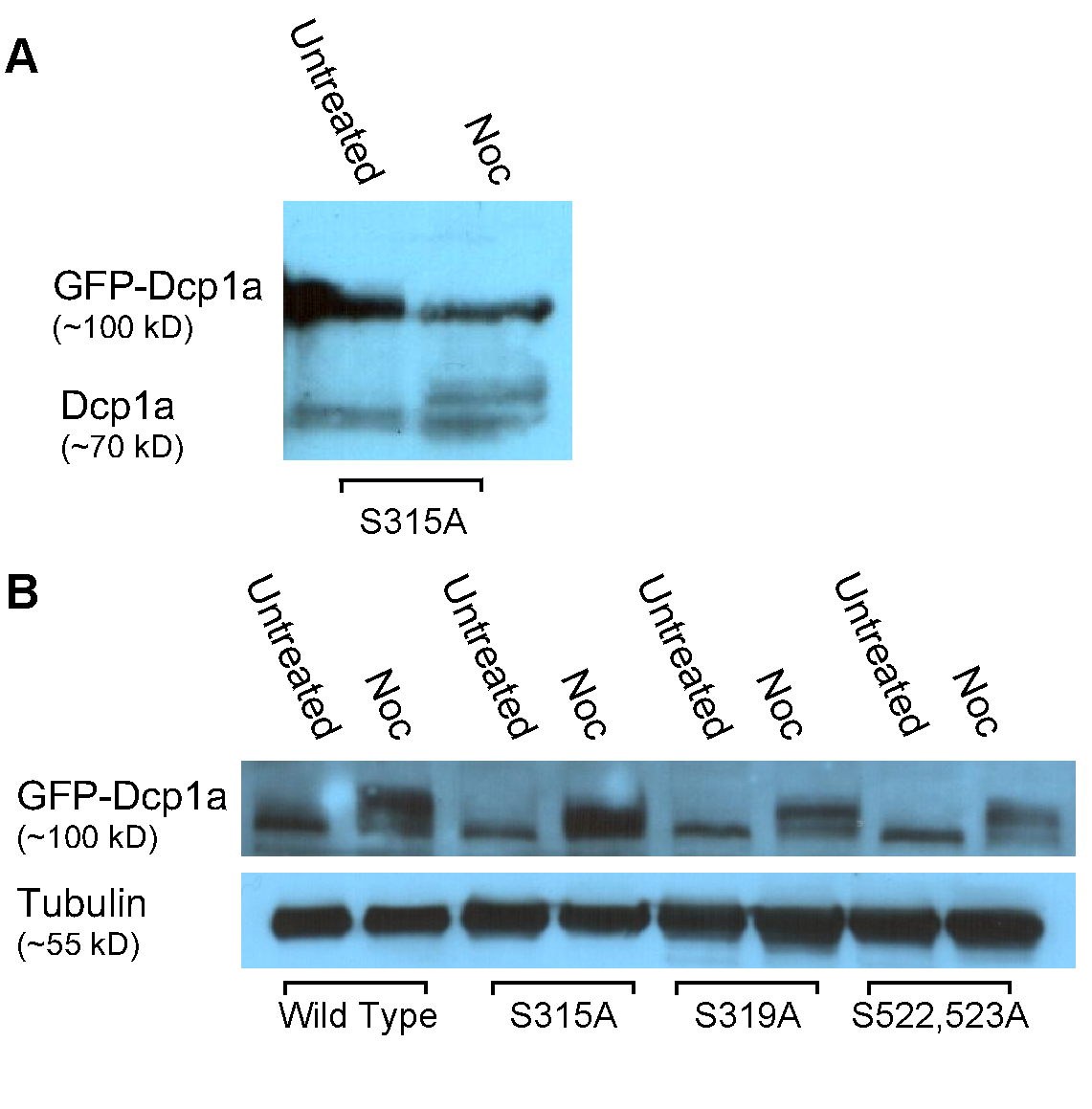

Supplement: Figure S3 — Mutation S315A reduces hyper-phosphorylation of GFP-Dcp1a. (A) No hyper-phosphorylation of hDcp1a S315A mutated protein was observed in mitotic cells (Noc) expressing GFP- hDcp1a S315A (100 kD) compared to the endogenous Dcp1a protein (70 kD) which did show hyper-phosphorylated Dcp1a bands. The blot was reacted with anti-Dcp1a. (B) The S319A and S522,523A mutated GFP-Dcp1a proteins showed prominent hyper-phosphorylation patterns compared to the S315A protein. Tubulin was used as a loading control. (TIF) [file pone.0049783.s005.tif]
